# Protecting Minors From Medical Malpractice Act: Implications for Otolaryngology–Head & Neck Surgery

**DOI:** 10.1002/lary.70402

**Published:** 2026-02-01

**Authors:** Shiven Sharma, Margo K. McKenna, Julina Ongkasuwan, Michael J. Brenner

**Affiliations:** ^1^ Department of Otolaryngology–Head and Neck Surgery Icahn School of Medicine at Mount Sinai New York USA; ^2^ University of Rochester Medical Center/Golisano Children's Hospital Rochester New York USA; ^3^ Department of Otolaryngology Baylor College of Medicine and Texas Children's Hospital Houston Texas USA; ^4^ Department of Otolaryngology–Head and Neck Surgery University of Michigan Medical School Ann Arbor Michigan USA; ^5^ Institute for Health Care Policy and Innovation University of Michigan Ann Arbor Michigan USA

**Keywords:** facial plastic and reconstructive surgery, medical malpractice, otolaryngology, patient safety, pediatrics, statute of limitations

*The Protecting Minors from Medical Malpractice Act of 2025* (S.209/H.R.653) establishes a federal private right of action against healthcare professionals who perform gender‐transition procedures on minors. It allows claims up to 30 years after majority for alleged physical, emotional, or psychological harm [1]. The Act defines gender‐transition procedures as puberty‐blocking drugs, cross‐sex hormones, or surgeries performed for the purpose of changing the body to align with a subjective gender identity that differs from an individual's biological sex. The *Act* is relevant to otolaryngology–head and neck surgery, where anatomical modification in childhood may have lasting effects on appearance, voice, and identity [2].

## 1 Discussion

The statutory language of the *Protecting Minors from Medical Malpractice Act of 2025* includes broad definitions of covered interventions and of harm, creating liability that potentially extends beyond the intended target [1]. Reconstructive pediatric surgeries can lead to delayed litigation, resulting in significant settlements [3, 4]. An otolaryngologic procedure performed in a pediatric patient without intent for gender transition could later be reinterpreted as influencing self‐perception of gender identity. Examples include laryngeal framework surgery, chondrolaryngoplasty, rhinoplasty, brow lift, otoplasty, and facial implants or reconstruction, since such procedures create anatomical changes that often persist into adulthood.

### 1.1 Extended Window for Liability

A 30‐year postmajority statute of limitations overrides state‐specific statutes of limitation and repose [1]. This unprecedented statute of limitations alters the risk environment for physicians even in states with shorter limitations periods. In New York, for example, medical malpractice claims must typically be filed within 2.5 years of the alleged negligence. For minors, the statute is tolled (paused) until age 18, but a 10‐year statute of repose bars actions after 10 years [5]. Thus, parents of a child injured at birth have until about age 10 to file a claim, far shorter than the 48‐year window the federal bill would allow. Other states have similar limitations that would be greatly extended under the *Act* [6, 7].

These inconsistencies between state and federal timelines introduce legal uncertainty. For otolaryngologists, procedures performed for therapeutic or reconstructive reasons may be reinterpreted decades later under new legal frameworks. Records may be incomplete, surgeons may change practices, and evolving standards add difficulty to legal defense. If these concerns disincentivize practitioners from offering procedures, overall access to pediatric otolaryngology care could decrease.

### 1.2 Patient Access and Best Practices

These access considerations are most pronounced for individuals seeking gender‐affirming facial plastic surgery or transgender voice services [8, 9], whereas implications for other procedures in otolaryngology are less clear. Legislative and insurance barriers, stigma, and rising liability can all constrain access to care across disciplines. Differences in payer coverage can also affect care access, both for procedures and continuity or comprehensiveness of care, although legal influences are unknown. The evolving medicolegal environment highlights the value of maintaining awareness of regulatory developments.

Otolaryngologists providing surgical care to minors should also be alert to how the *Act* can influence liability with both gender‐transition procedures and those procedures that could be construed as identity altering. Informed consent and accompanying documentation should reflect the intent of procedures and consideration of long‐term outcomes. Documentation should specify the medical indication, the risks and benefits, and the guardian's understanding. If applicable, there may also be a role for specifying whether the procedure was intended for gender‐affirming purposes. In academic institutions, it is also appropriate to record resident involvement and supervision. A summary of potential strategies appears in Table 1.

**TABLE 1:** Best practices for counseling and documentation around procedural intent.

Strategy	Implementation details
Enhanced informed consent	Document the medical indications, describe the expected functional and aesthetic outcomes, and specify whether the intervention addresses a medical condition or serves a gender‐affirming purpose.
Long‐term record retention	Verify institutional policies to securely retain operative notes, pre‐ and post‐operative photos, and signed consents for extended periods.
Standardized terminology	Align diagnostic and procedural language with established coding and clinical guidelines to avoid ambiguity around goals of surgical procedures.
Professional society guidance	Engage with specialty societies to develop guidance and terminology that ensures clarity in procedural intent, and communicate these distinctions to policymakers.

## 2 Conclusion

*The Protecting Minors from Medical Malpractice Act of 2025* alters the malpractice landscape by extending the legal timeline to decades beyond the closure points established under state law. These considerations are relevant for otolaryngologists whose surgical practice involves developmental anatomy, aesthetics, or combined functional/aesthetic procedures, including reconstructive procedures that could influence long‐term psychosocial outcomes. The *Act*'s definitions and statute of limitations reinforce the value of aligning clinical decisions, documentation, and counseling practices with both ethical standards and shifting federal liability frameworks.

## Funding

The authors have nothing to report.

## Disclosure

The Icahn School of Medicine at Mount Sinai (ISMSS) IRB noted an exemption for this manuscript as only publicly available and deidentified data were included in the study.

## Conflicts of Interest

The authors declare no conflicts of interest.

## Data Availability

The data that support the findings of this study are available from the corresponding author upon reasonable request.
